# Repeatability and reliability of exploratory behavior in proactive and reactive zebrafish, *Danio rerio*

**DOI:** 10.1038/s41598-018-30630-3

**Published:** 2018-08-14

**Authors:** Matthew R. Baker, Alexander C. Goodman, Jonathan B. Santo, Ryan Y. Wong

**Affiliations:** 1 Department of Biology, University of Nebraska Omaha, Omaha, Nebraska USA; 2 Department of Psychology, University Nebraska Omaha, Omaha, Nebraska USA

## Abstract

Behavioral responses to novel situations often vary and can belong to a suite of correlated behaviors. Characteristic behaviors of different personality types (e.g. stress coping styles) are generally consistent across contexts and time. Here, we compare the repeatability and reliability of exploratory behaviors between zebrafish strains selectively bred to display contrasting behavioral responses to stressors that represent the proactive-reactive axis. Specifically, we measure exploratory behavior of individual fish in an open field test over five weeks. We quantified the stationary time, average swimming speed and time spent by a fish in the center area. We found a number of strain differences for each behavioral measure. Stationary time was the most repeatable and reliable measure for assessing proactive-reactive behavioral differences. Reactive zebrafish generally showed the highest reliability and repeatability of exploratory behavior compared to proactive zebrafish and a separate wild caught strain. Given the increased interest in the evolutionary consequences and proximate mechanisms of consistent individual differences, it will be important to continue to investigate how different selective pressures may influence expression of stress coping styles and their effects on the consistency of an animal’s behavior.

## Introduction

Animals are frequently faced with a variety of stressors to their survival and reproductive efforts and typically employ behavioral and physiological responses to overcome them. While an individual’s behavioral response has been thought to be labile in variable environments, studies show that it can be consistent. Consistency of behavioral and physiological responses have both a between and within-individual component where the response in one context is often predictive of the animal’s response in a disparate context and across time^1–5^. In response to stressors, many studies document consistent differences between individuals across contexts in behaviors like aggression, boldness and sociability but the temporal component within the same context has not been thoroughly examined^4–8^.

Across many taxa there are two alternative correlated suites of behavioral and physiological responses to stressors known as proactive and reactive stress coping styles^9,10^. Proactive individuals are characterized by actively engaging stressors, having a feed-forward memory process, low glucocorticoid stress response, and low behavioral flexibility. In contrast, reactive individuals are more sensitive to environmental cues with reduced exploration of novel environments, a higher glucocorticoid stress response, and higher behavioral flexibility^10^. Both coping styles represent adaptive responses to environmental challenges and are maintained within a population due to fitness trade-offs in a variable environment^1,5,11–13^. Selective pressures such as predation and immune challenge can constrain behavioral variation within a population and strengthen correlations between traits, thereby facilitating correlated behaviors and physiology^14–16^.

Selectively bred strains of proactive and reactive behavioral phenotypes are often used to investigate the proximate mechanisms of stress coping styles and estimate heritability^17^. Few studies have investigated repeatability (between-individual consistency) and reliability (within-individual consistency) of behaviors in stress coping styles across multiple time points. Studies looking at aggressive behavior of selectively bred mice (*Mus musculus*) suggest that proactive individuals with low behavioral flexibility show greater reliability across trials than reactive individuals^18,19^. Similarly, proactive great tits (*Parus major*) establish more rigid routines during an open field test (OFT) and novel object test while reactive birds showed a higher degree of behavioral plasticity and lower reliability^20^. While some studies report conflicting results in teleosts^21,22^, selectively bred reactive trout (*Oncorhynchus mykiss*) display higher behavioral flexibility when a novel object was introduced during a feeding task^23^. Despite these observations, the repeatability and reliability of stress coping styles across time has not been well-established. Further, it is also not well understood how artificial selection may affect repeatability and reliability of behavior.

Exploratory behavior in a novel environment can be used to assess the magnitude of a stress response and categorize an individual into a stress coping style. Variation in exploratory behavior is widely studied and often utilizes the well-established OFT^24–26^. Within-individual variation of exploratory and other behaviors over time are influenced by factors such as prior experience, age, and motivation^7,8^. To investigate within-individual consistency across time, studies have used the reliability estimate, which measures the predictability of an animal’s performance on a measured variable over time relative to others within a population (0 = no predictability, 1 = perfect predictability). In human and animal personality studies, reliability estimates tend to range between 0.7–0.85^27^. A frequently used estimate to measure consistent differences between individuals is repeatability. The repeatability of a behavior is defined as the intraclass correlation coefficient and is calculated as the ratio of between-individual variance and the sum of between- and within-individual variance. Overall, exploratory behavior in a novel environment is repeatable in many avian, rodent, and teleost species with repeatability values ranging from 0.2–0.5^28–30^. In addition to exploratory behavior, other behaviors such as thigmotaxis and movement speed have been commonly used to assess the magnitude of behavioral stress response and could also be indicative of an individual’s stress coping style^31,32^. Thigmotaxis and movement speed are generally repeatable like exploratory behavior^33^. It is unknown which behavior (exploratory behavior, thigmotaxis, or movement speed) is most suitable for use as an indicator of an individual’s stress coping style.

Zebrafish (*Danio rerio)* are a promising teleost system to understand the causes and consequences of correlated behavioral variation^6,34,35^. Both wild and laboratory strains of zebrafish display the proactive and reactive stress coping styles, and have distinct and heritable genetic architectures^11,16,36–42^. Proactive zebrafish are typically dominant and have higher reproductive success^43,44^. We have previously shown that selectively bred proactive and reactive zebrafish strains show consistent behavioral differences across a variety of contexts between the strains^41^. Additionally, artificial selection of exploratory behavior will constrain morphological evolution and glucocorticoid responses^31,45^. While the consistency of exploratory behavior has not been extensively studied in individual strains of proactive and reactive zebrafish, several studies have suggested that other boldness, aggression, and locomotor behaviors are generally consistent across contexts and time, and are influenced by selective pressures^33,46–48^. Thus, zebrafish can provide unique insights into underlying mechanisms of behavioral variation in coping with stress and subsequently how variation can be constrained by selective forces acting on populations.

In this study, we examined repeatability and reliability of three estimates of exploratory behavior in individual zebrafish during weekly OFT behavioral assays over five weeks. We used wild-derived strains selectively bred to display proactive and reactive stress coping styles to determine if (i) exploratory behavior is stable across time, (ii) repeatability and reliability measures differ between the stress coping style strains, and (iii) selectively bred strains are more repeatable or reliable than a separate wild caught population. By measuring variation of behavior within and between individuals, we can gain insight into factors contributing to the emergence and maintenance of stress coping styles in different populations^11,49^. As correlated traits constrain behavioral plasticity, they also have large implications regarding underlying genetics and heritability, which can lead to shifts in evolutionary trajectories^50–52^.

## Methods

### Subjects

We used three different zebrafish (*Danio rerio*) strains: wild caught (WC), high stationary behavior (HSB), and low stationary behavior (LSB). Wild caught fish were imported from North Bengal, India through a commercial supplier (Nebraska Aquatic Supply, Omaha, Nebraska, USA) and housed in the laboratory for 21 months before testing. The HSB and LSB strains were selected for stationary behavior (i.e. exploratory behavior) in an open field test and were 10 generations removed from a wild caught population from Gaighata in West Bengal, India^41^. The HSB and LSB strains display behaviors across multiple different behavioral assays, glucocorticoid responses, and morphology consistent with the reactive and proactive stress coping styles, respectively^31,41,45,53,54^. Additionally, HSB and LSB strains differ in neurotranscriptome profiles^54,55^. Females of both strains exhibit higher stationary time than males in an open field test^41,56^. We tested 28 individuals from LSB (N = 12 males, 16 females) and 27 each from HSB (N = 13 males, 14 females) and WC strains (N = 13 males, 14 females). LSB and HSB individuals were 13 months post-fertilization when testing began. Fish were individually housed in 3-liter tanks throughout the period of experiments on a recirculating water system (Pentair Aquatic Eco-Systems) using UV and solid filtration. Water temperature was set at 27 °C. Fish were kept on a 14:10 L/D cycle and fed twice a day with Tetramin Tropical Flakes (Tetra, USA). Morning feedings were prior to experiments on testing days.

### Experiments

To test the repeatability and reliability of exploratory behavior, we used the open field test following established procedures^26,41,54^. Briefly, a plexiglass testing arena (30 × 30 × 10 cm) was filled with 4 L of system water. Animals were individually placed in the arena and video-recorded for 5 min. Each fish was tested once a week for five consecutive weeks between 8 and 10 h in the morning. The video recordings were analyzed with Noldus EthoVision XT (Noldus XT, Wageningen, Netherlands). For each fish, we quantified three estimates of exploratory behavior: stationary time, average swimming speed and time spent in the center. The subject was considered stationary if it was moving less than 0.5 cm/s and the center zone was defined as the 15 × 15 cm zone in the center of the chamber. We digitally measured standard length of each fish at end of the five weeks. There was a significant effect of strain on standard length (F_2,79_ = 35.84 p < 0.01). The WC strain (3.27 ± 0.05 cm) was significantly larger than the HSB (2.84 ± 0.04 cm; p < 0.01) and LSB lines (2.78 ± 0.05 cm; p < 0.01). HSB and LSB lines did not significantly differ in standard length (p = 0.42). Females (3.03 ± 0.04 cm) were significantly larger than males (2.90 ± 0.04 cm; F_1,79_ = 6.79 p = 0.01). All testing experiments were approved by the Institutional Animal Care and Use Committee of University of Nebraska at Omaha/University of Nebraska Medical Center (17-070-00-FC, 17-064-08-FC) and were performed in accordance with the relevant guidelines and regulations.

### Statistics

Reliability of exploratory behavior across time and between-strain differences were tested using a repeated measures general linear model (GLM) in SPSS (Version 24). Sex and strain were included as between-subjects variables and standard length was controlled for by including it as a covariate. Since the assumption of sphericity was violated for each of the three exploratory behaviors we applied the Greenhouse-geisser correction. This did not change any statistical conclusions, therefore we only reported the uncorrected model. For the post-hoc comparisons of the estimated marginal means of the three estimates of exploratory behavior, we applied a Benjamini-Hochberg correction to reduce the likelihood of type I errors^57^.

To assess behavioral variation among strains, we used multilevel structural equation modeling in M-plus statistical analysis software^58^. This allowed us to control for shared associations between the behaviors in a single model, and more importantly test for differences in the variability within- and between-individuals across strains. First, we began with an unconditional model to assess the variability at each level (e.g. within- and between-individual variability). Next, the covariances between all the behaviors were included at each level. Then, we split the models by strain (LSB, HSB and WC) and constrained every part of the model in a step-wise fashion to ascertain which sources of variability were significantly different across the strains (first at the within-individual level and then the between-individual level). A constraint was considered to have worsened the model based on a significant chi-square test (*p* < 0.05). Any significantly worsening constraint reflects a difference in the estimates between strains.

Repeatability was defined as the intraclass correlation coefficient (R), which was calculated as the ratio of between-individual variance and the sum of between- and within-individual variance^59^. We calculated R based on variance components estimated from the multilevel structural equation model. Based on several literature meta-analyses^60,61^ we describe repeatability values as follows: low repeatability R ≤ 0.2; moderate repeatability 0.2 < R < 0.4; and high repeatability R ≥ 0.4. Reliability measures were estimated as the inter-trial reliability measure in SPSS (Version 24). Values > 0.8 were considered highly reliable and indicate that individuals maintained rank order across the five weeks of testing. Of note, repeatability and reliability values are deemed significantly different from a comparison value if they do not fall within that comparison value’s 95% confidence interval. All statistical tests were two-tailed, and were conducted with an alpha level of 0.05.

## Results

### Repeatability and reliability of exploratory behavior across time

There was a significant between-subjects effect of strain for stationary time (F_2,79_ = 15.75 p < 0.01, Table 1). The WC strain spent significantly less time stationary than the LSB (p < 0.01) and HSB strains (p < 0.01). The LSB strain also spent significantly less time stationary than the HSB strain (p = 0.03), as expected. Further, there was high repeatability for stationary behavior in the HSB (R = 0.71) and LSB (R = 0.56) strains, while the WC strain was moderately repeatable (R = 0.28). All three repeatability values were significantly different from each other for stationary time (Table 2). There was a significant effect of strain on swimming speed (F_2,79_ = 3.37 p = 0.04, Table 1). The WC strain swam significantly faster than the HSB (p = 0.02), but not the LSB strain (p = 0.26). HSB and LSB lines did not significantly differ in mean swimming speed (p = 0.13). Further, all strains showed high repeatability for mean swimming speed (WC: R = 0.40; HSB: R = 0.59; LSB: R = 0.55). The HSB strain had significantly higher repeatability than the WC, but not the LSB strain for mean swimming speed (Table 2). For time spent in the center there was an effect of strain (F_2,79_ = 40.73 p < 0.01, Table 1).The WC strain spent significantly less time in the center than the LSB (p < 0.01) and HSB strains (p < 0.01). The LSB strain spent significantly more time in the center zone than the HSB strain (p = 0.01). The HSB (R = 0.46) strain was significantly more repeatable than the WC (R = 0.21) and LSB (R = 0.10) strains. The LSB strain’s repeatability was not significantly different from zero (Table 2). All other between-individual factors were not significant.

**Table 1:** Results of repeated measures GLM for behavioral estimates across time and post hoc tests. For post-hoc results, estimated marginal mean (EMM) values with different superscript letters indicate significant differences.

	Stationary Time	Mean Swimming Speed	Time in Center
	F_(df)_	F_(df)_	F_(df)_
Within-Subjects Effects			
Week	1.23_(4, 300)_	0.46_(4, 300)_	2.37 _(4, 300)_
Week*Standard Length	1.23_(4, 300)_	0.37_(4, 300)_	**2**.97*_(4, **300)**_
Week*Strain	1.89_(8, 300)_	0.87_(8, 300)_	**2**.95*_(8, **300)**_
Week*Sex	0.26_(4, 300)_	0.67_(4, 300)_	1.16 _(4, 300)_
Week*Strain*Sex	0.98_(8, 300)_	1.39_(8, 300)_	0.60 _(8, 300)_
Between Subjects Effects			
Intercept	0.34_(1, 75)_	2.18_(1, 75)_	1.03 _(1, 75)_
Standard Length	0.19_(1, 75)_	0.37_(1, 75)_	0.29 _(1, 75)_
Strain	**11**.04*_(2, **75)**_	**3**.16*_(2, **75)**_	**24**.76*_(2, **75)**_
Sex	0.03_(1, 75)_	1.65_(1, 75)_	0.23 _(1, 75)_
Strain*Sex	0.42_(2, 75)_	0.06_(2, 75)_	2.99 _(2, 75)_
Post-Hoc Tests (LSD)	EMM	EMM	EMM
HSB	204.80^a^	3.12^a^	174.53^a^
LSB	148.05^b^	4.64^a,b^	242.31^b^
WC	56.92^c^	6.14^b^	62.59^c^

Note: *p < 0.05.

**Table 2:** Repeatability values (intraclass correlation (95% confidence intervals)) of each behavior by strain (Abbreviations: HSB, high stationary behavior; LSB, low stationary behavior; WC, wild caught).

	N	Stationary Time	Mean Swimming Speed	Time in Center
HSB	27	0.71 (0.59–0.83)	0.59 (0.44–0.74)	0.46 (0.29–0.64)
LSB	28	0.56 (0.40–0.73)	0.55 (0.38–0.72)	0.10 (−0.08–0.27)
WC	27	0.29 (0.10–0.47)	0.40 (0.22–0.58)	0.21 (0.03–0.38)

**Figure 1:**
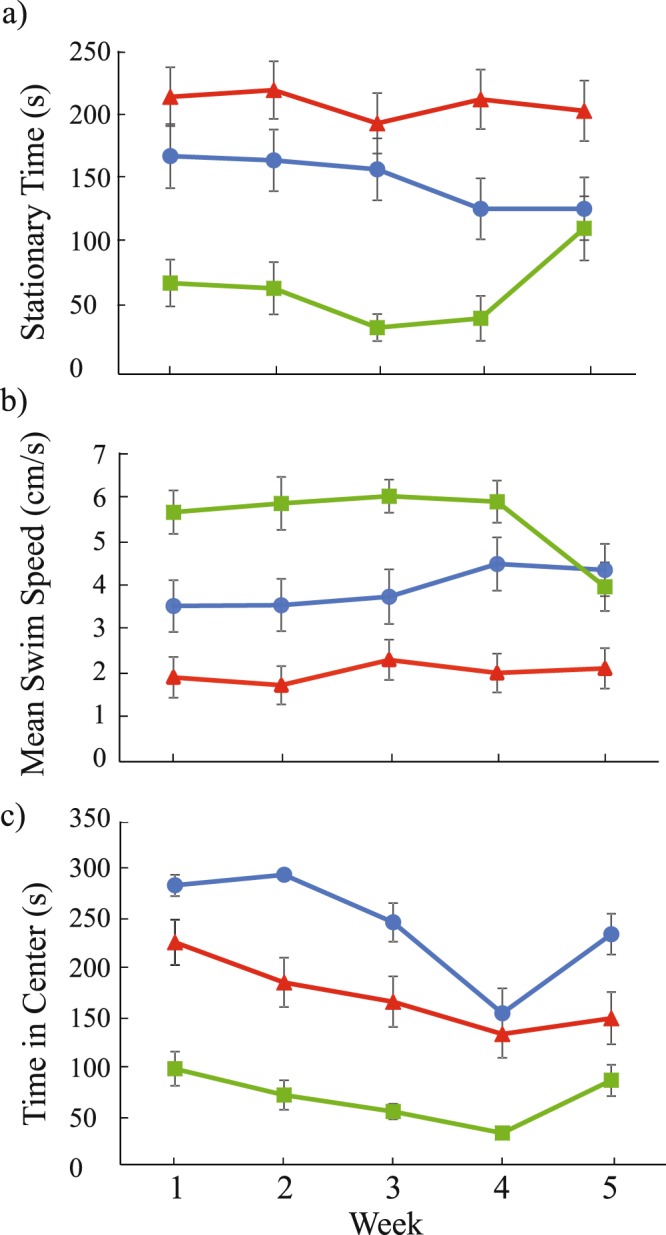
Stationary time (**a**), mean swimming speed (**b**), and time in center (**c**) performance across five weeks of testing for each zebrafish strain. Points represent weekly means (±1 SE). The blue circle, red triangle, and green square points represent the low stationary behavior (LSB), high stationary behavior (HSB), and wild-caught (WC) strains, respectively. There was a significant strain*time effect for time spent in the center.

There were significant interaction effects of week*strain (F_4, 300_ = 2.97 p < 0.01) and week*standard length (F_4,300_ = 2.95 p = 0.02) for time spent in the center (Table 1, Fig. 1). Further, the HSB strain was significantly more reliable (Reliability = 0.85) than the LSB (Reliability = 0.58) and WC (Reliability = 0.57) strains for time spent in the center (Table 3). There were no significant effects of week, nor any significant interaction effects for stationary time (all *p* > 0.05; Fig. 1). The HSB strain was significantly more reliable (Reliability = 0.94) compared to WC (Reliability = 0.70), but not LSB (Reliability = 0.86) fish for stationary time (Table 3). There were no significant effects of week, nor any significant interaction effects for mean swimming speed (all *p* > 0.05; Fig. 1). The HSB strain (Reliability = 0.90) was significantly more reliable than the WC (Reliability = 0.80), but not the LSB fish (Reliability = 0.85) for mean swimming speed (Table 3).

**Table 3:** Reliability values (95% confidence intervals) of each behavior by strain (Abbreviations: HSB, high stationary behavior; LSB, low stationary behavior; WC, wild caught).

	N	Stationary Time	Mean Swimming Speed	Time in Center
HSB	27	0.94 (0.89–0.97)	0.90 (0.82–0.95)	0.85 (0.73–0.92)
LSB	28	0.86 (0.76–0.93)	0.85 (0.74–0.92)	0.58 (0.27–0.78)
WC	27	0.70 (0.47–0.85)	0.80 (0.64–0.89)	0.57 (0.24–0.78)

### Behavioral variation across strains

The unconditional model revealed that between 50.03–60.78% of the variability in mean swimming speed, stationary time, and time spent in the center was at the between-individual level. Not surprisingly, this represented a significant proportion of within- and between-individual variability for all three estimates of exploratory behavior (*p* < 0.05). The resulting model provided adequate fit (χ^2^_(3)_ = 1.15, *p* > 0.05, CFI = 1.00, RMSEA < 0.01, SRMR_(within)_ < 0.01, SRMR_(between)_ = 0.10). After constraining the variability at the within-individual level, a number of differences emerged (Table 4). Specifically, the HSB strain had significantly less variability in both mean swimming speed and stationary time at the within-individual level. Meanwhile, the WC strain had significantly less variability in time spent in the center. At the between-individual level, the LSB strain had significantly higher variability in mean swimming speed. Time spent in the center was significantly more variable in the HSB strain. Lastly, the WC strain had significantly lower variability in stationary behavior.

**Table 4:** Multi-level modeling differences in the sources of variability as a function of strain.

	LSB		HSB		WC	
	Est. (S.E.)	z^*p*^	Est. (S.E.)	z^*p*^	Est. (S.E.)	z^*p*^
SPEED (within variance)	4.95 (0.70)	7.04*	**2**.**31** (**0**.**53)**	**4**.33*	4.95 (0.70)	7.04*
STATIONARY (within variance)	6622.00 (855.67)	7.74*	**3463**.**60** (**890**.**36)**	**3**.89*	6622.00 (855.67)	7.74*
CENTER (within variance)	9580.55 (1280.39)	7.48*	9580.55 (1280.39)	7.48*	**4071**.**24** (**1109**.**10)**	**3**.67*
SPEED (between variance)	**6**.**09** (**1**.**19)**	**5**.11*	3.30 (0.59)	5.61*	3.30 (0.59)	5.61*
STATIONARY (between variance)	8544.47 (1365.97)	6.26*	8544.47 (1365.97)	6.26*	**2649**.**69** (**818**.**20)**	**3**.24*
CENTER (between variance)	1057.41 (360.01)	2.94*	**8273**.**17** (**1732**.**80)**	**4**.77*	1057.41 (360.01)	2.94*

Note: Values in bold reflect significant differences in the variability across strains. *p < 0.05.

## Discussion

An animal’s tendency to explore during unpredictable or risky situations is indicative of its stress coping style. It is unclear whether behavioral traits of a stress coping style are repeatable and reliable. Here, we found effects of strain, but not sex or standard length, on within- and between-individual variation for three estimates of exploratory behavior. All three behavioral estimates were generally repeatable and reliable. Overall, the selectively bred strains of zebrafish showed higher repeatability and reliability values compared to the WC fish. Further, the HSB strain showed remarkably high repeatability and reliability for all three behaviors, and had significantly less within-individual variability compared to LSB and WC strains for both swimming speed and stationary behavior. Stationary time was the most repeatable behavioral measure and was consistent across time. Time spent in the center zone showed the greatest variability across weeks compared to stationary time and swimming speed. While several studies have found sex-specific behavioral variation of mating and aggressive behaviors^46,47,60^, we did not observe any sex differences in exploratory behavior.

High exploratory behavior in a novel environment is characteristic of the proactive stress coping style^9,10^. Previous studies have demonstrated that selection on exploratory behavior can strengthen correlations between other stress coping behaviors in other contexts, glucocorticoid levels, and morphology^31,41,45^. Here, we show that artificial selection can also constrain behavioral variation in populations across time. HSB fish showed significantly higher stationary behavior than LSB animals, which is consistent with previous studies^41,54^. While the WC strain was moderately repeatable (R = 0.29), both of the LSB (R = 0.56) and HSB (R = 0.71) strains showed high repeatability values for stationary time (Table 2). There was no effect of time, and selectively bred fish maintained rank order across the five time points (Reliability > 0.8, Table 3). Further, the HSB line showed significantly less within-individual variability compared to LSB and WC strains (Table 4). Emergence of consistent individual differences in the presence of selective pressures are also documented in other species^14,15^. Field crickets (*Gryllus integer*) exposed to a common bacterial pathogen showed increased repeatability in their tendency to explore a novel environment^15^. Similarly, boldness and aggression behaviors were correlated in wild sticklebacks (*Gasterosteus aculeatus*) only after exposing the population to predation^14^. In great tits, correlation between exploratory behavior and stress physiology emerged through selectively bred proactive and reactive birds but not wild individuals^62^. These studies suggest that selection can influence expression of repeatability and reliability by potentially placing survival costs on individuals. Predation has frequently been identified as one of the strongest ecological pressures that can influence the repeatability and reliability of animal behavior^11,16,47^. We speculate that by selecting for an ecologically relevant behavioral response to a stressor in the HSB and LSB lines within the lab, it may have been simulating selection of behavioral responses to predation in the wild. Low exploratory behavior may be adaptive and directly selected for in environments with high predation. This could explain why the HSB fish showed the highest repeatability and reliability values for a majority of the behavioral estimates. Altogether, this indicates that artificial selection may act similarly to natural selection and increase the repeatability and reliability of behavior.

Increased thigmotaxis in many species is indicative of higher stress levels and an aversion to being exposed in the center of a novel environment^63,64^. Despite being a measure of stress and type of exploratory behavior, thigmotaxis has not been commonly used to predict an animal’s stress coping style. We observed that the repeatability of time in the center in the HSB, WC, and LSB strains was high, low, and not repeatable, respectively (Table 2). Similarly, HSB fish maintained rank order (Reliability = 0.85), whereas LSB and WC fish did not (Reliability < 0.6; Table 3). The WC strain had the highest amount of thigmotaxis (indicating high stress levels) but spent the least amount of time stationary (suggestive of proactive stress coping style). The combination of low repeatability and reliability for all but the HSB strain and conflicting behavioral interpretations in the WC strain suggest that thigmotaxis may not be an accurate proxy measure for proactive-reactive tendencies.

It should be noted that the WC individuals were imported from North Bengal, India, which is a different location than the founding animals used to generate the HSB and LSB strains (West Bengal, India). The local ecological factors that might have contributed to shaping the WC animals’ behavior are not known. It is possible that some population differences or behavioral correlations may only emerge under certain local environmental conditions^16,46,65^. Several studies that investigated wild zebrafish populations found that predation and water flow can explain population differences in behavioral correlations between boldness, aggression and activity^16,46^. While these studies examined between-population differences, similar patterns have been observed within populations across time in collared flycatcher birds (*Ficedula albicollis*). Behavioral correlations appeared and disappeared across years that coincided with changes in the density and age composition of the bird population, which could reflect changes in resource availability^65^. Our results suggest that the ecological pressures acting on the WC population shaped different behavioral patterns compared to those resulting from artificial selection.

Studies show that a faster swimming speed and larger body size are suggestive of a proactive coping style^31,66^. There were no significant differences between the HSB and LSB strains in standard length or mean swimming speed. Although a previous study showed that the LSB strain swims faster and has a larger caudal region compared to the HSB strain, this was examined using morphometrics and within a startle-response paradigm^31^. Measuring standard length does not allow for inferring size of specific body areas (e.g. caudal region). In our study we also measured average swim speed over a five minute period within the open field test and did note evoke a startle-response. All three strains were highly repeatable for average swimming speed (Table 2), similar to wild trout (*Salmo trutta*)^33^. There was no effect of time across weeks (Table 1), and all strains maintained rank order (Table 3) for mean swimming speed. Further, HSB fish showed higher repeatability and reliability values than the WC strain, as well as significantly lower within-individual variability compared to LSB and WC strains (Table 4). Similar to stationary time, the selection force shaping the behavior may have a greater effect on the HSB than the LSB strain. It is possibly related to environmental contexts where high or low exploratory behavior would be adaptive and selected for. However, with no significant difference in swimming speed between the HSB and LSB strains, other measures such as maximum velocity or acceleration should be considered in future studies for assessing stress coping style differences.

Previous studies suggest that proactive individuals with low behavioral flexibility and rigid behavior patterns are more consistent than reactive individuals^18–20^. Our results show the opposite pattern. HSB individuals had significantly less within-individual variability in stationary time and mean swimming speed across five weeks (Table 4). Further, the HSB strain had the highest repeatability and reliability values for each of the behavioral measures (Tables 2 and 3). It is possible that the conflicting observations with prior studies can be attributed to different focal behavior measured or the strength of selection on the behavior. For example, in two mice lines bidirectionally selected for divergent aggressive behaviors towards conspecifics, proactive individuals showed higher consistency of aggressive behaviors compared to reactive individuals^18,19^. In trout bidirectionally selected for divergent cortisol reactivity to confinement stress, reactive trout displayed higher behavioral flexibility across trials when a novel object was introduced to a feeding task^23^. The current study used zebrafish strains bidirectionally selected for opposing exploratory behavior in response to a novelty stressor. Thus, the behavioral flexibility trait of stress coping styles appear to vary across stressor types (e.g. conspecific, novel object, novel environment) and selectively bred traits (e.g. behavioral and physiological).

It is also important to be able to separate out changes in consistency across time from those that could be due to changes in contexts between assay time points^7^. In studies examining repeatability and reliability within a social context such as aggressive behaviors^18,19^ and predator inspection^67,68^, it is difficult to ensure consistency of behaviors and motivation of live stimulus animal across trials. Thus, the context the focal animal experiences may subtly vary across testing periods and make it difficult to understand if results are due to stimulus animal behavioral state or within-individual variation over time. In using the OFT we removed potential confounds of varying contexts over time and therefore are confident we measured within-individual variation. Even when these changing contexts are taken into account, other internal developmental factors can also influence the repeatability and reliability of behavior. Despite using a similar methodology as the current study, selectively bred proactive great tit birds were more consistent over time in exploratory behavior relative to the reactive birds^20^. It is noteworthy that the two assay time points were across developmentally distinct periods (once in juveniles and once in adults), which has been shown to influence repeatability^60^. In the current study, all zebrafish were sexually mature adults at the time of testing and were assayed over five weeks, which could explain the inconsistency of our findings.

There are many key considerations when estimating repeatability and reliability of animal behavior. Often studies have estimated repeatability by using two measurements for each individual, which can overlook any behavioral changes that may occur over longer periods of time or multiple observations^69^. This is especially important given that a central assumption of stress coping styles is that a behavioral phenotype is maintained over time, despite the observation that animal behavior can be very labile over small durations^61^. Here, we found that stationary time was the most repeatable and reliable estimate of exploratory behavior over five repeated observations. Additionally, the artificially selected proactive and reactive strains showed higher repeatability and reliability values compared to the wild caught population. This suggests that in populations under high levels of selection, a single to a few measurements for the examined behaviors can be a sufficient representation of that individual’s behavior. While increasing the number of repeated trials allow for more robust repeatability estimates, short inter-trial intervals can lead to habituation or other forms of associative learning. These types of learning can have confounding effects on estimates of repeatability and reliability^7,70^. If animals habituate during repeated measurements, there is weaker construct validity of assessing consistency of stress coping behaviors. We found no evidence of habituation in our study with a one week intertrial interval (Fig. 1). Overall, behavioral repeatability and reliability metrics are important for insight into selective pressures that may support development of stress coping styles. Future studies should test the fitness consequences associated with high or low behavioral consistency. Additionally, while stress coping styles have been well documented to have a genetic basis, the neural and molecular mechanisms are only beginning to be explored. Artificial bidirectional selection can serve as a complementary approach to understanding proximate mechanisms underlying consistent individual differences in stress coping behaviors.

## Electronic supplementary material

Dataset 1

## Data Availability

All data generated or analyzed during this study are included in this published article and its Supplementary Information files.
